# Tissue-Specific Strategies of the Very-Long Chain Acyl-CoA Dehydrogenase-Deficient (VLCAD^−/−^) Mouse to Compensate a Defective Fatty Acid β-Oxidation

**DOI:** 10.1371/journal.pone.0045429

**Published:** 2012-09-14

**Authors:** Sara Tucci, Diran Herebian, Marga Sturm, Annette Seibt, Ute Spiekerkoetter

**Affiliations:** Department of General Pediatrics and Neonatology, University Childreńs Hospital, Duesseldorf, Germany; Cinvestav-IPN, Mexico

## Abstract

Very long-chain acyl-CoA dehydrogenase (VLCAD)-deficiency is the most common long-chain fatty acid oxidation disorder presenting with heterogeneous phenotypes. Similar to many patients with VLCADD, VLCAD-deficient mice (VLCAD^−/−^) remain asymptomatic over a long period of time. In order to identify the involved compensatory mechanisms, wild-type and VLCAD^−/−^ mice were fed one year either with a normal diet or with a diet in which medium-chain triglycerides (MCT) replaced long-chain triglycerides, as approved intervention in VLCADD. The expression of the mitochondrial long-chain acyl-CoA dehydrogenase (LCAD) and medium-chain acyl-CoA dehydrogenase (MCAD) was quantified at mRNA and protein level in heart, liver and skeletal muscle. The oxidation capacity of the different tissues was measured by LC-MS/MS using acyl-CoA substrates with a chain length of 8 to 20 carbons. Moreover, in white skeletal muscle the role of glycolysis and concomitant muscle fibre adaptation was investigated. In one year old VLCAD^−/−^ mice MCAD and LCAD play an important role in order to compensate deficiency of VLCAD especially in the heart and in the liver. However, the white gastrocnemius muscle develops alternative compensatory mechanism based on a different substrate selection and increased glucose oxidation. Finally, the application of an MCT diet over one year has no effects on LCAD or MCAD expression. MCT results in the VLCAD^−/−^ mice only in a very modest improvement of medium-chain acyl-CoA oxidation capacity restricted to cardiac tissue. In conclusion, VLCAD^−/−^ mice develop tissue-specific strategies to compensate deficiency of VLCAD either by induction of other mitochondrial acyl-CoA dehydrogenases or by enhancement of glucose oxidation. In the muscle, there is evidence of a muscle fibre type adaptation with a predominance of glycolytic muscle fibres. Dietary modification as represented by an MCT-diet does not improve these strategies long-term.

## Introduction

Mitochondrial β-oxidation is one of the most important processes for cellular energy production. The first oxidation step of long-chain fatty acids (C14–20) is catalyzed by the very long-chain acyl-CoA dehydrogenase (VLCAD). Fatty acids with chain lengths of C6-C12 and C10-C16 are also oxidized by medium-chain acyl-CoA dehydrogenase (MCAD) and long-chain acyl-CoA dehydrogenase (LCAD), respectively. All three enzymes display partial overlapping substrate specificity and share extensive homology [1].

VLCAD-deficiency (VLCADD) is the most common long-chain fatty acid oxidation disorder with a regional incidence between 1∶30,000 and 1∶100,000 [2]–[5]. Molecular heterogeneity in VLCADD corresponds to heterogeneous clinical phenotypes [6]. Symptoms occur in organs and tissues with a high metabolic rate such as liver, heart and skeletal muscle with different severity and age of onset [4]. Situations of increased energy demand i.e. prolonged fasting, infectious illnesses or physical exercise, when the organism mostly relies on fatty acid β-oxidation, may trigger the development of clinical symptoms and may cause serious metabolic derangement. Typical symptoms are cardiomyopathy, hepatopathy, hypoketotic hypoglycemia, muscle weakness and episodic rhabdomyolysis [7], [8]. As part of long-term treatment and during catabolic situations, the application of sufficient carbohydrates and medium-chain triglycerides (MCT) is recommended to bypass the first step of β-oxidation catalyzed by VLCAD supplying tissues and organs with the required energy.

The VLCAD^−/−^ mouse represents an excellent model for the investigation of VLCADD as it presents with a very similar clinical phenotype than humans [9]. Under non-stressed conditions, VLCAD^−/−^ mice display an altered calcium homeostasis [10] as well as changes in key genes and proteins of fatty acid metabolism in liver, heart and brown adipose tissue [11]–[13]. Moreover, as occurring in humans, fasting, cold exposure and intensive physical exercise trigger the development of symptoms resulting in the accumulation of long-chain acylcarnitines, hypoglycaemia, hepatopathy and skeletal myopathy [14]–[18].

Similar to many patients with VLCADD, VLCAD^−/−^ mice remain asymptomatic over long periods of time. In order to identify the involved compensatory mechanisms, we assess the expression at mRNA and protein level of the mitochondrial dehydrogenases MCAD and LCAD. Because of the chain-length specificity only towards C4 and C6 acyl-CoA, short-chain acyl-CoA dehydrogenase (SCAD) was not included in this study. The investigations are conducted in heart, liver and skeletal muscle of one year old VLCAD^−/−^ mice. Mitochondrial proliferation in response to a defective fatty acid oxidation is measured in the tissues by citrate synthase activity. Furthermore, the effect of VLCAD deletion on the turnover rate of the other mitochondrial acyl-CoA dehydrogenases is quantified by measuring the oxidation capacity of the different tissues using a variety of acyl-CoA substrates with a chain length of 8 to 20 carbons. In white skeletal muscle glycolytic activity and the distribution of muscle fibre types is determined. Finally, the assumed functional compensation due to dietary intervention is analyzed in the same manner in heart, liver and skeletal muscle of VLCAD^−/−^ mice supplemented over one year with an MCT-modified diet.

## Materials and Methods

### Animals

Experiments were performed on fourth- to fifth-generation intercrosses of C57BL6+129sv VLCAD genotypes. Littermates served as controls and genotyping of mice was performed as described previously [11]. Groups consisting of 5–7 mice, one year old, were investigated under well-fed, non-fasting conditions. Mice were sacrificed under well-fed conditions by CO_2_ asphyxiation. Liver, heart and white skeletal muscle (*gastrocnemius muscle*) were rapidly removed and immediately frozen in liquid nitrogen.

All animal studies were performed with the approval of the Heinrich-Heine-University Institutional Animal Care and Use Committee and in accordance with the Committees' guidelines. Approval of the Landesamt für Natur, Umwelt und Verbraucherschutz (LANUV) (File number: 8.87–50.10.34.09.072).

### Diet composition and supplementation

After weaning, at approx. 5–7 weeks of age, mice of each genotype were divided in two groups and fed with different diets for one year. The first group received a normal purified mouse diet containing 5% crude fat in form of LCT, corresponding to 12% of metabolizable energy as calculated with Atwater factors (ssniff® EF R/M Control, ssniff Spezialdiäten GmbH, Soest, Germany). The second group was fed with a diet corresponding as well to 12% of total metabolizable energy. Here 4.4% from a total of 5% fat was MCT (Ceres®MCT-oil, basis GmbH, Oberpfaffenhofen, Germany) while the remaining 0.6% was derived from soy bean oil to provide the required essential long-chain fatty acids. Both diets based on purified feed ingredients contained the same nutrient concentration as follows: 94.8% dry matter, 17.8% crude protein (N x 6.25), 5% crude fat, 5% crude fibre, 5.3% crude ash, 61.9% nitrogen free extract, 36.8% starch, 14.8% dextrin and 11% sugar. The detailed fatty acid composition of the diets was previously reported [19]. In both diets the carbohydrate and protein contents corresponded to 69% and 19% of metabolizable energy, respectively. All mice groups received water *ad libitum*.

### Tissue homogenates and protein expression

Tissues were homogenized with Cellytic MT Buffer (Sigma-Aldrich, Steinheim, Germany) in the presence of 1mg*ml^−1^ protease inhibitors and centrifuged at 4°C and 16,000 g for 10 min to pelletize any cell debris. The clear supernatant was immediately used for the enzyme assays or stored at –80°C. Protein concentration of tissue homogenates was determined using the BSA method as described previously [20].

Protein expression in the different tissues was performed by western blot analysis. 10–20 µg protein homogenate from tissue lysate were separated on a gradient (4–12%) SDS polyacrylamide gel and transferred to nitrocellulose. Detection was carried out with anti MCAD antibodies (monoclonal mouse, Mitoscience) and anti LCAD antibodies (polyclonal mouse, Abnova) used at a 1∶1000 and 1∶2000 dilution, respectively. Anti GAPDH (monoclonal mouse, Applied Biosystems) was used as a loading control at 1∶4000. HRP-conjugated secondary antibodies were used at 1∶5000. Signals were detected and quantified with a LAS 3000 Luminescent Image Analyzer (Fujifilm, Germany).

### Citrate synthase and catalase assay

Citrate synthase assay was performed in triethanolamine-HCl buffer (100 mM, pH 8.0), 300 μM acetyl-CoA, 100 μM 5,5′-dithiobis-(2-nitrobenzoic acid) and 8 µg tissue homogenate. The assay mixture was preincubated 10 min at 25 C before addition of 500 μM oxaloacetate as substrate. The activity was determined photometrically by measuring the absorbance at 412 nm for 2 min. Catalase activity was measured fluorometrically by the production of the highly fluorescent oxidation product resorufin [21], [22].

### Acyl-CoA oxidation measured by LC-MS/MS

Acyl-CoA oxidation rate was measured with a variety of saturated acyl-CoA substrates as reported previously [23]. Octanoyl (C8:0)-CoA, decanoyl, (C10:0)-CoA, dodecanoyl (C12:0)-CoA, tetradecanoyl (C14:0)-CoA, palmitoyl (C16:0)-CoA, stearoyl (C18:0)-CoA, and arachidoyl (C20:0)-CoA were purchased from Sigma (Steinheim, Germany). Acyl-CoAs were detected by LC-ESI-MS/MS in positive ionization mode according to the published literature (ter Veld 2009) using following modifications: spray voltage 4 kV, extractor 2 V, RF lens 1 V, source temperature 120°C, desolvation temperature 325°C, cone gas flow 60 L/h, desolvation gas flow 700 L/h, collision energy 35 V, dwell time 0.1 seconds. Following fragmentation ions including their cone voltages were used for the selected reaction monitoring (SRM) mode: C8-CoA-assay (894.2>387.2 for C8:0-CoA, 892.2>385.2 for C8:1-CoA, 910.2>403.2 for C8:OH-CoA, cone 25 V); C10-CoA-assay (922.5>415.2 for C10:0-CoA, 920.2>413.2 for C10:1-CoA, 938.2>431.2 for C10:OH-CoA, cone 35 V); C12-CoA-assay (950.2>443.2 for C12:0-CoA, 948.2 > 441.2 for C12:1-CoA, 966.2>459.2 for C12:OH-CoA, cone 40 V); C14-CoA-assay (978.2>469.2 for C14:0-CoA, 976.2>469.2 for C14:1-CoA, 994.2>487.2 for C14:OH-CoA, cone 45 V); C16-CoA-assay (1006.2>499.2 for C16:0-CoA, 1004.2>497.2 for C16:1-CoA, 1022.2>515.2 for C16:OH-CoA, cone 45 V); C18-CoA-assay (1034.2>527.2 for C18:0-CoA, 1032.2>525.2 for C18:1-CoA, 1050.2>543.2 for C18:OH-CoA, cone 45 V); C20-CoA-assay (1062.2>555.2 for C20:0-CoA, 1060.2>553.2 for C20:1-CoA, 1078.2>571.2 for C20:OH-CoA, cone 45 V). The experiments were carried out on a Quattro Micro tandem mass spectrometer (Waters, UK) coupled with a 2795 Alliance HPLC system (Waters, UK). The chromatographic conditions were as follows: Phenomenex C18(2) Luna column (100 mm x2.0 mm x3 µm), C18 security guard cartridges (4×3.0 mm), aqueous phase consisted of 0.01 % ammonium hydroxide while the organic phase of acetonitrile for C14-, C16-, C18- and C20-CoA-products and of acetonitrile/2-propanol (v/v; 1/1) for C8-, C10-, and C12-CoA-products. Aqueous phase percentages of the total mobile phase were 60% for C8-, 40% for C10-, 60% for C12-, C14-, C16- and 70% for C18-and C20-CoA-products. 10 µL of the assay mixture was injected and the products were eluted isocratically over 5 minutes at room temperature. Data acquisition and analysis were performed by using the MassLynx Version 4.1 software (Waters, UK). Peak integration and calculation were carried out with QuanLynx 4.1 software (Waters, UK).

### Real Time PCR analysis

Total liver RNA was isolated with the RNeasy mini kit (Qiagen, Hilden Germany). Forward and reverse primers for β-actin (BC138614), peroxisome proliferative activated receptor gamma coactivator 1 alpha (*PGC1a*; BC066868), pyruvate dehydrogenase kinase isoenzyme 4 (*PDK4* NM_013743), medium-chain acyl-CoA dehydrogenase (*ACADM*; NM_007382), long-chain acyl-CoA dehydrogenase (*ACADL*; NM_007381.3), peroxisome proliferative-activated receptor alpha (*PPARα*; NM_011144.6), troponin I skeletal, slow 1 (*Tnni1*; NM_021467), troponin I, skeletal, fast 2 (*Tnni2*; NM_009405), troponin C, cardiac/slow skeletal (*Tnnc1*; NM_009393), troponin C2, fast (*Tnnc2*; NM_009394) and troponin T1, skeletal, slow (*Tnnt1*; BC141142), annotated in **Table** **1**, were designed with the FastPCR program (R. Kalendar, Institute of Biotechnology, Helsinki). Real Time PCR was performed in a single step procedure with the QuantiTect SYBR Green^TM^ RT-PCR (Qiagen, Hilden, Germany) on an Applied Biosystems 7500 Sequence Detection System in Micro Amp 96-well optical reaction plates capped with MicroAmp optical caps (Applied Biosystems, Foster City, CA, USA) as previously described [24]. The values in all samples were normalized to the expression level of the internal standard.

**Table 1:** Primer used for RT-PCR analysis.

Gene	Forward 5′→3′	Reverse 5′→3′
*ACADM*	GAAAGTTGCGGTGGCCTTGG	AAGCACACATCATTGGCTGGC
*ACADL*	GGGAAGAGCAAGCGTACTCC	TCTGTCATGGCTATGGCACC
*PPARα*	GCTCTGTCATCACAGACACC	CCTTCACATGCGTGAACTCC
*PGC1*α	CAACATGCTAAGCCAAAC	GGAGTTAGGCCTGCAGTTCC
*PDK4*	CCTGTCAGAGTTTGTAGACAC	CAAAGGCATCTTGGACTACTG
*Tnni1*	ACGAACAGGTGCAGCCTTGC	GCTCCGAGAGGTAACGCACC
*Tnni2*	CATGGACCTGAGGGCCAACC	ACTGGATGTTGGGTGCTTCC
*Tnnc1*	AGGATGGCTGCATCAGCACC	GAAGTCCACTGTGCCACTGC
*Tnnc2*	GCCAGCAACCATGACGGACC	CCTCCTTTGGTGGGTGTCTGC
*Tnnt1*	CAAACCCAGCCGTCCTGTGG	GCACGTGATGAGCGTCTGC
β-actin	TAGGCACCAGGGTGTGATGG	CTCCATGTCGTCCCAGTTGG

*ACADM,* medium-chain acyl-CoA dehydrogenase; *ACADL,* long-chain acyl-CoA dehydrogenase; *PGC1a,* peroxisome proliferative activated receptor gamma coactivator 1 alpha; *PDK4,* pyruvate dehydrogenase kinase isoenzyme 4; *PPARa,* peroxisome proliferative activated receptor alpha; *Tnni1*, troponin I skeletal, slow 1; *Tnni2*, troponin I, skeletal, fast 2; *Tnnc1*, troponin C, cardiac/slow skeletal ; *Tnnc2*, troponin C2, fast and *Tnnt1,* troponin T1, skeletal, slow.

### Statistical analysis

All reported data are presented as means ± standard error of the mean (SEM). *n* denotes the number of animals tested. Analysis for the significance of differences was performed using Student's t-tests for paired and unpaired data. To test the effects of the two variables, diet and genotype, two-way analysis of variance (ANOVA) with Bonferroni post-test was performed. (GraphPad Prism 5, GraphPad Software, San Diego California USA). Differences were considered significant if *p*<0.05.

## Results

### Clinical phenotype

As shown in Table 2, under normal mouse diet no significant differences were observed in mean body weights between the WT and VLCAD^−/−^ group (24.65±0.74 vs. 26.70±0.93 g). After one year supplementation with MCT, WT mice displayed a slight increase in mean body weight (27.25±1.52 g) although this increment was not significant. We could not observe a similar trend in VLCAD^−/−^ mice under the same dietary regimen (26.50±1.12 g).

**Table 2:** Mean body weight of WT and VLCAD^−/−^ mice fed either with LCT or MCT diet.

	WT	VLCAD−/−
LCT	26.65±0.74	26.7±0.93
MCT	27.65±1.52	26.50±1.12

Body weights are expressed in gram (g) and are given as mean of mean ± SEM (*n* = 5–7).

### Tissue mRNA expression of the β-oxidation genes *ACADM* and *ACADL*

**Figure 1:**
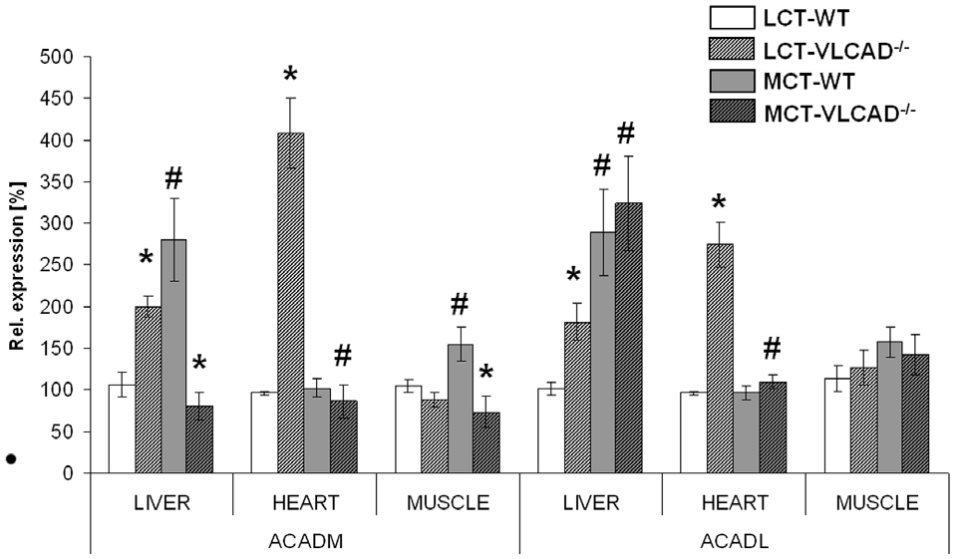
mRNA expression of different dehydrogenases in mice fed either with an LCT or MCT diet. LCT, long-chain triglyceride; MCT, medium-chain triglyceride; *ACADM*, medium-chain acyl-CoA dehydrogenase and *ACADL,* long-chain acyl-CoA dehydrogenase.Values are mean of mean ± SEM (*n* = 5–7). * indicates significant differences between WT and VLCAD^−/−^ mice within a diet group. # indicates significant differences between WT or VLCAD^−/−^ mice under different dietary conditions. Values are considered significantly different if p<0.05 (Two way ANOVA and Student's t-test).

To determine the distribution of *ACADM* and *ACADL*, we examined the expression pattern of these genes in the different tissues of VLCAD^−/−^ mice by using RT-PCR. As shown in **Fig.** **1** **,** under control diet we observed in the VLCAD^−/−^ mice a strong up-regulation of *ACADM* and *ACADL* in liver and heart tissue as compared to WT mice, suggesting significant compensation of long-chain fatty acid oxidation by MCAD and LCAD. A similar increase in gene expression was not assessed in the skeletal muscle. Because an MCT diet is mainly composed by C8 and C10 fatty acids [19], we expected a strong up-regulation of *ACADM* and *ACADL* after MCT supplementation for one year. Indeed, MCT effects could be seen in the liver of VLCAD^−/−^ mice and WT mice, whereas the heart and skeletal muscle did not show any enhancement on gene expression. However, *ACADM* was not up-regulated in liver of VLCAD^−/−^ mice. In accordance with our previously published data regarding the elongation of medium-chain fatty acids as result of an MCT diet [14], [17], [19], we propose that the elongated fatty acid is oxidize as demonstrated by an increase in *ACADL* expression but cannot be shortened in sufficient amount due to deletion of VLCADD.

### Western blot analysis

**Figure 2:**
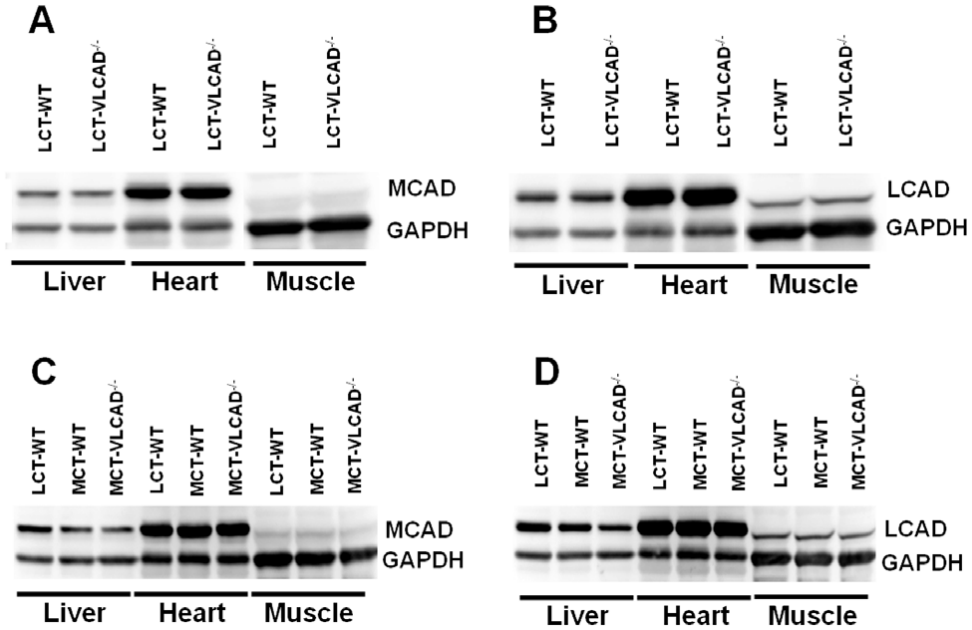
Western blot analysis of different dehydrogenases in mice fed either with LCT or MCT diet. LCT, long-chain triglyceride; MCT, medium-chain triglyceride; *ACADM*, medium-chain acyl-CoA dehydrogenase; *ACADL,* long-chain acyl-CoA dehydrogenase; *GAPDH,* Glyceraldehyde 3-phosphate dehydrogenase. Each *lane* represents pooled homogenates and contains 10 µg of liver / heart lysates and 20 µg of gastrocnemius muscle. Pools are prepared with protein homogenates of *n* = 5–7 animals.

To establish whether an up-regulation of *ACADM* and *ACADL* at mRNA level corresponds to an increased protein synthesis, we performed western blot analysis in liver, heart and skeletal muscle homogenates. As shown in **Fig.** **2A–B**, the expression of both proteins did not differ between WT and VLCAD^−/−^ mice under normal mouse diet. In contrast to the expression at mRNA level after MCT supplementation the protein content of MCAD and LCAD was not increased in both genotypes (**Fig.** **2C–D**). In fact, we observed a slight although not significant reduction of MCAD and LCAD protein content in the liver and skeletal muscle of WT and VLCAD^−/−^ mice.

### Citrate synthase activity (CS)

**Figure 3:**
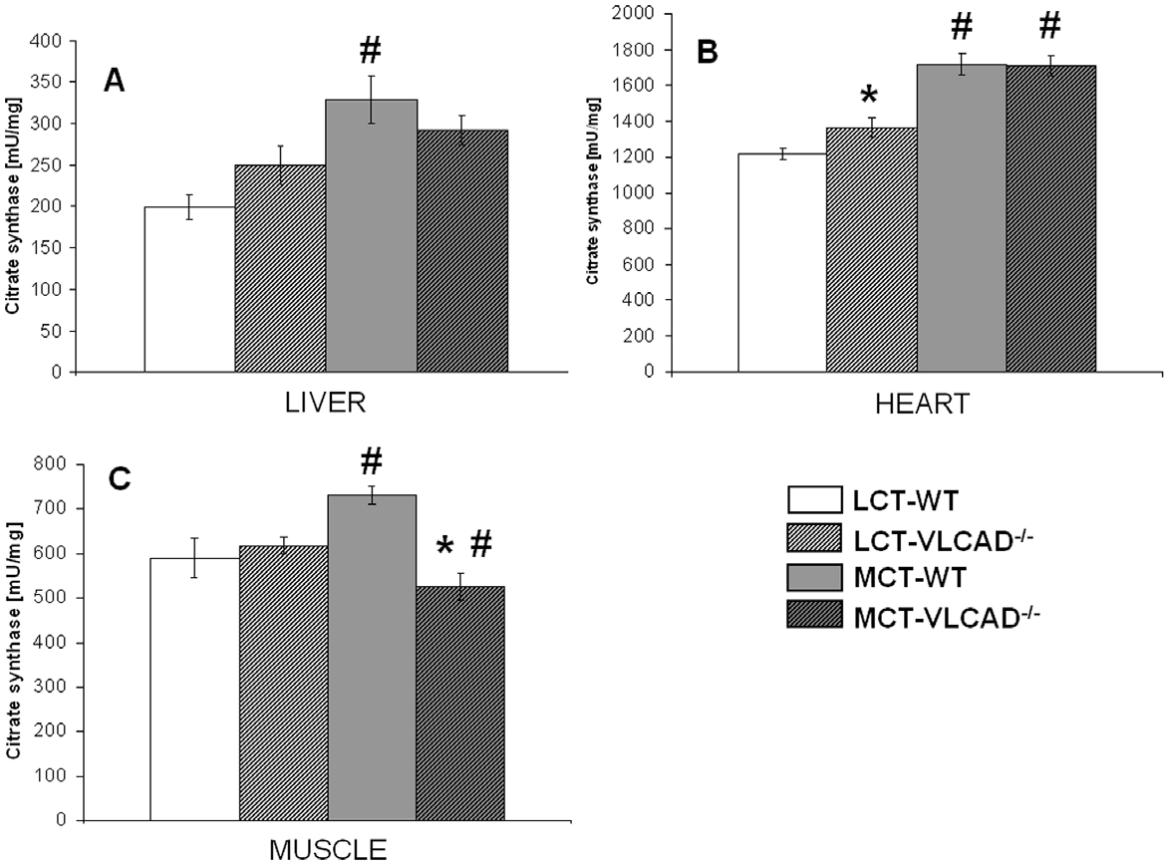
Citrate synthase activity in WT and VLCAD^−/−^ mice fed either with LCT or MCT diet. Tissues tested are (A) liver, (B) heart and (C) skeletal muscle. Specific activity is expressed in mU/mg protein and is given as mean of mean ± SEM (*n* = 5–7).* indicates significant differences between WT and VLCAD^−/−^ mice within a diet group. # indicates significant differences between WT or VLCAD^−/−^ mice under different dietary conditions. Values are considered significantly different if p<0.05 (Two way ANOVA and Student's t-test).

The measurement of the citrate synthase activity under a normal diet only revealed differences in heart homogenates with a turnover rate significantly higher in VLCAD^−/−^ mice as compared to WT mice (1214.34±32.48 vs. 1364.48±53.71 mU/mg) indicating an increase in mitochondrial number. A higher energy supply as provided by MCT led to a significant increase in CS specific activity in the liver of WT mice and a slight although not significant increase in the liver of VLCAD^−/−^ mice (**Fig.** **3A**). The increment was even more pronounced in heart tissue of both genotypes as we observed a strong induction of CS activity, as shown in **Fig.** **3B** **.** Indeed, upon MCT diet we measured specific cardiac activity values of 1717±56.6 and 1710±54.8 mU/mg in WT and VLCAD^−/−^ mice, respectively, indicative of mitochondrial proliferation. A similar effect was also observed in the muscle homogenate of WT mice, in contrast to a decreased CS activity in muscle of VLCAD^−/−^ mice as they are not able to metabolize the fatty acids supplied by MCT likely due to ready elongation (**Fig.** **3** **.C**). This finding suggests other strategies for energy production than fatty acid oxidation in skeletal muscle.

### Effects of VLCAD-deficiency and MCT supplementation on the oxidation of different acyl-CoA substrates in different tissues

In order to gain more insight into the oxidation capacity of liver, heart and skeletal muscle in VLCAD^−/−^ mice and to evaluate the role of other enzymes with overlapping substrate specificities we analyzed the oxidation of saturated acyl-CoAs with a chain length of C8 to C20.

Liver

**Figure 4:**
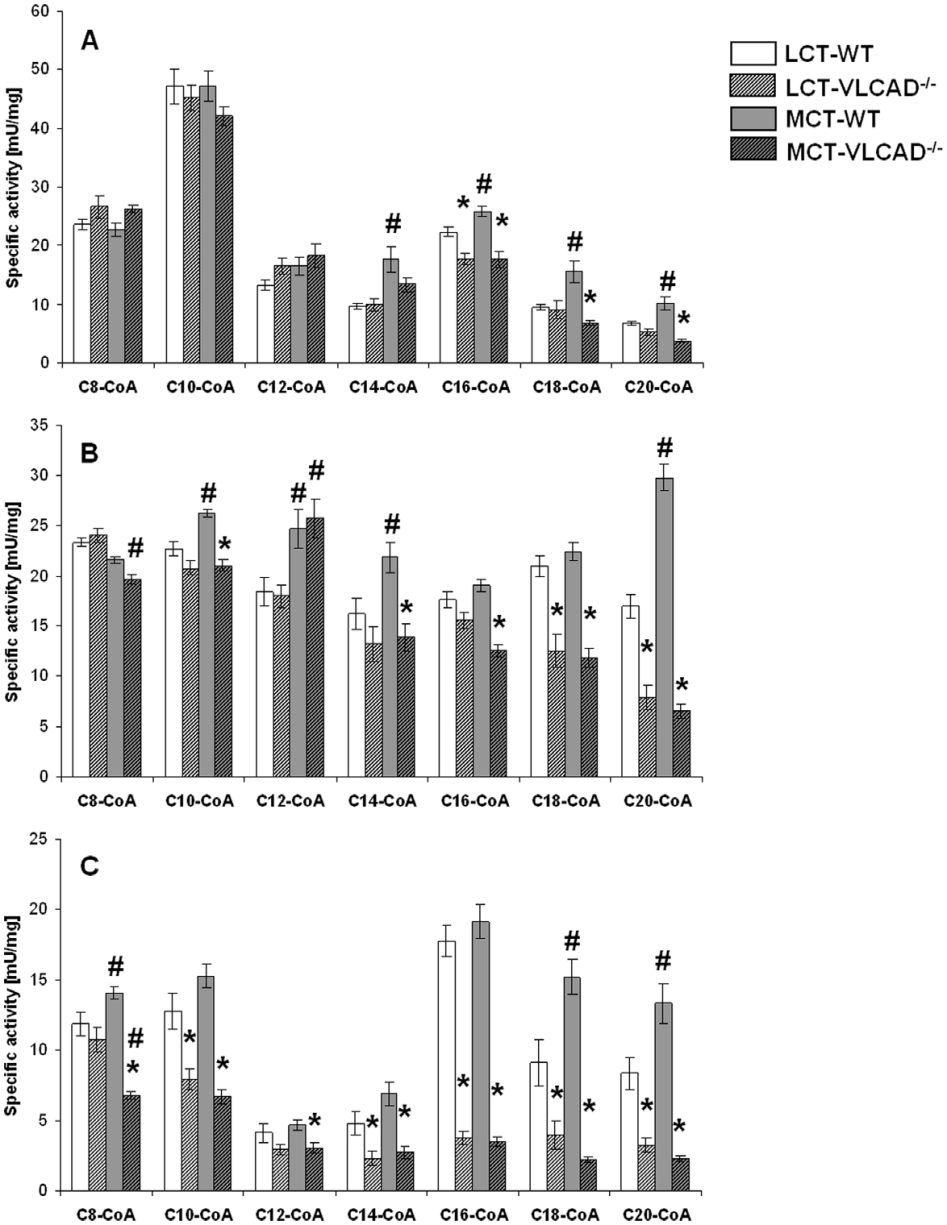
Oxidation rates in WT and VLCAD^−/−^ mice fed either with LCT or MCT diet. Oxidation rate was determined with straight-chain acyl-CoA substrates. Tissues tested were (A) liver, (B) heart and (C) skeletal muscle. Oxidation is expressed in mU/mg protein and is given as mean of mean ± SEM (*n* = 5–7).* indicates significant differences between WT and VLCAD^−/−^ mice within a diet group. # indicates significant differences between WT or VLCAD^−/−^ mice under different dietary conditions. Values are considered significantly different if p<0.05 (Two way ANOVA and Student's t-test).

In the liver, the highest turnover rate was observed with C10-CoA as substrate independently of the dietary regimen. Under regular diet conditions VLCAD^−/−^ mice showed a significantly lower turnover rate as compared to WT mice with C16-CoA as substrate, however, sill about 80% of the rate in WT (C16-CoA; 22.4±0.8 vs 17.7±1.03 mU/mg). These data suggest a strong contribution of C16-CoA oxidation by other enzymes than VLCAD. All the other substrates were oxidized at the same rate as in WT mice. After supplementation with an MCT diet over one year, WT mice exhibited a significantly increased oxidation of long-chain acyl-CoA (C14-C20) compared to mice under control diet which may be attributed to the increased lipogenesis and elongation of long-chain fatty acids (**Fig.** **4A**) [14], [17], [19]. However, no effects occurred with respect to the substrate oxidation in VLCAD^−/−^ mice upon MCT diet.

#### Heart

The substrate oxidation pattern of heart homogenates slightly differed from that of the liver displaying overall lower substrate affinity to medium-chain acyl-CoA (C8-C10). Upon a control diet the turnover rate of most of the acyl-CoA was comparable in both genotypes as shown in **Fig** **4B**. Surprisingly, C16-CoA oxidation was not reduced in VLCAD^−/−^ mice as compared to WT mice suggesting complete compensation by LCAD. However, we observed a significant decrease in oxidation capacity in VLCAD^−/−^ mice up to 40% and 53% with C18-CoA and C20-CoA, respectively, as substrate. Supplementation with MCT for one year does not have significant effect on acyl-CoA oxidation in VLCAD^−/−^ mice. Interestingly, both genotypes presented with an increased turnover rate of C12-CoA with MCT.

#### Muscle

The very low content of fatty acid oxidation enzymes in skeletal muscle as demonstrated by western blot analysis was reflected by a reduced oxidation capacity in muscle homogenate in parallel, in contrast to the other tissue lysates. As shown in **Fig.** **4C** **,** the turnover rate in VLCAD^−/−^ mice was for nearly all acyl-CoA substrates significantly lower as compared to WT mice suggesting little compensation by other dehydrogenases than VLCAD. This effect was very striking when C16-CoA was used as substrate, as the WT mice displayed a high activity of 17.7±1.07 vs 3.8±0.46 mU/mg in VLCAD^−/−^ mice. Although MCT mainly comprises C8 and C10 fatty acids [19] that can be metabolized by VLCADD, VLCAD^−/−^ mice fed with MCT for one year still displayed a further reduction of C8-CoA oxidation as compared to untreated mice suggesting that MCT is not an alternative energy source long-term.

### Metabolic and morphological adaptation of skeletal muscle in VLCAD^−/−^mice

**Figure 5:**
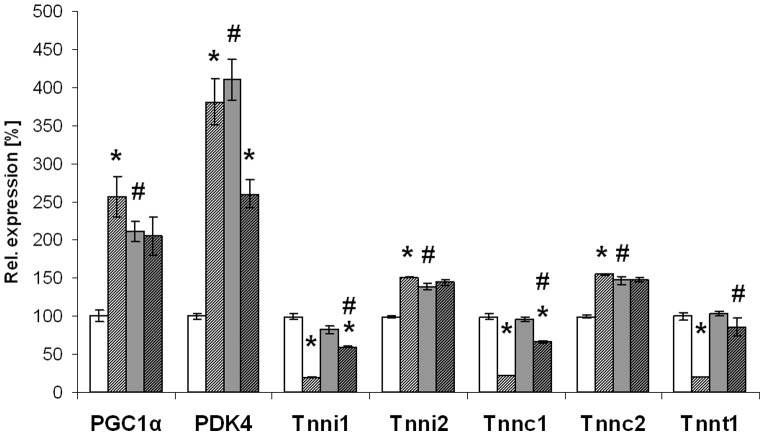
Substrate selection and gene expression of different muscle fibre types. LCT, long-chain triglyceride; MCT, medium-chain triglyceride; *PGC1a,* peroxisome proliferative activated receptor gamma coactivator 1 alpha; *PDK4,* pyruvate dehydrogenase kinase isoenzyme 4; *Tnni1,* troponin I skeletal, slow 1; *Tnni2,* troponin I, skeletal, fast 2; *Tnnc1,* troponin C, cardiac/slow skeletal; *Tnnc2*, troponin C2, fast; *Tnnt1*, troponin T1, skeletal, slow. Values are mean of mean ± SEM (*n* = 5–7). * indicates significant differences between WT and VLCAD^−/−^ mice within a diet group. # indicates significant differences between WT or VLCAD^−/−^ mice under different dietary conditions. Values are considered significant if p<0.05 (Two way ANOVA and Student's t-test).

Because of the low turnover rate of long- and medium-chain acyl-CoAs in skeletal muscle of VLCAD^−/−^ mice we tested the expression of genes regulating energy homeostasis and glucose metabolism (**Fig.** **5**). Under an LCT diet we observed an up-regulation of *PGC1α* and *PDK4* in VLCAD^−/−^ mice suggesting energy deficiency. Since *PGC1α* regulates the switch to the oxidative type I muscle fibres [25], [26] we further investigated whether this occurred in VLCAD^−/−^ mice. In fact, the troponin genes *Tnni1*, *Tnnc1* and *Tnnt1* coding for the slow-twitch oxidative type I fibres were strongly down-regulated, in contrast to the up-regulation of *Tnni2*, *Tnnc2* expressing the glycolytic type II fibres. Upon MCT diet, also WT mice displayed a significant up-regulation of *PGC1α* and *PDK4* but no differences in the expression of the troponin genes. In contrast, VLCAD^−/−^ mice presented with a slighter but still significant up-regulation of *Tnni1*, *Tnnc1* and *Tnnt1* as compared to a regular LCT diet suggesting an initial switch in the promotion of a predominantly oxidative fibre type I.

## Discussion

In this study we demonstrate that VLCAD^−/−^ mice compensate deficiency of VLCAD with different strategies in the different tissues. Because mutants develop symptoms mainly during catabolic situations and are asymptomatic during normal life [14]–[16], [18], [19], [27], [28], we proposed to observe functional compensatory mechanisms [29], [30] such as overexpression of dehydrogenases with overlapping substrate specificity for medium and long-chain fatty acyl-CoAs. In that, VLCAD^−/−^ mice displayed a strong up-regulation of the mitochondrial acyl-CoA dehydrogenase (ACAD) mRNA transcripts in the liver and in the heart suggestive of a signal response due to energy deficiency, while the expression in the skeletal muscle was unaffected. However, despite the pronounced up-regulation at mRNA level all analyzed tissues of VLCAD^−/−^ mice do not express MCAD and LCAD at protein level at a higher extent, indicating that the net amount of ACAD is not influenced by deficiency of VLCAD. Of interest was the finding that the ACAD content strongly differs within the tissues. With special regard to the heart and skeletal muscle, the very striking difference in ACAD expression can be explained by the distinct physiological function of muscular fibre types [31]–[34] linked to meet the specific energy requirement of the organs either by fat or glucose [35]–[37]. In accordance with the measurement of the citrate synthase activity, considered a suitable parameter for the mitochondrial content [38], [39], the high ACAD expression in the heart reflects a higher efficiency in energy supply by fat and a better adaptation to tissue-specific energy demand [35] whereas the white skeletal muscle mostly relies on glycolysis that occurs in cytoplasm.

LCAD protein which displays substrate overlap with VLCAD is expressed in mice at high extent [40]. Our results on the turnover rate with long-chain acyl-CoA substrates demonstrate that liver and heart largely benefit from the high oxidation activity of LCAD, although this enzyme is reported to catalyze mostly unsaturated acyl-CoAs rather than saturated chains [40], [41]. The contribution of ACAD9 is minor since this enzyme is recently described to possess different physiological functions than fatty acid oxidation [42], [43]. In addition to that, the expression of PPARα and the specific activity of the peroxisomal catalase display no genotype or diet dependent up-regulation in all analyzed organs indicating that under resting condition the peroxisomal support to acyl-CoA oxidation is not of relevance (**Fig.** **S1**). The redirection of long-chain acyl-CoA oxidation *via* LCAD is an efficient mechanism to avoid accumulation of toxic long-chain acylcarnitines and energy deficiency especially under well-fed resting conditions, as analyzed in this study. Nevertheless, in heart homogenate the oxidation capacity with regard to substrate affinity is mismatched between both genotypes. Indeed, the WT mice displayed the highest substrate affinity to C18-CoA while VLCAD^−/−^ mice to C16-CoA. A difference in the pattern of substrate affinity based on acyl-CoA chain length between LCAD and VLCAD has been already described for other species [41] and our results in mice are also supported by the different accumulating long-chain acylcarnitines in LCAD^−/−^ and VLCAD^−/−^ mice, respectively [40].

In strong contrast, skeletal muscle seems to be less sustained by LCAD contribution and there is evidence of an alternative compensatory strategy. A previous study with young VLCAD^−/−^ mice showed that the oxidation rate for C16-CoA was 50% of the corresponding littermates [14]. However, in one year old VLCAD^−/−^mice the turnover rate for C16-CoA and also for medium-chain acyl-CoA is strongly reduced. The concomitant up-regulation of genes encoding for type II glycolytic fibres suggests that the muscle fibres undergo metabolic and structural modification leading to increased glucose oxidation. Despite the switch toward a different substrate to compensate VLCAD deletion, the very high expression of PGC1α is indicative of metabolic stress accompanied by inactivation of glycolysis in parallel, as reflected by the up-regulation of PDK4 [44]. These observations suggest that the compensatory mechanism based on adaptation in the substrate selection in the long run may not be effective.

Medium-chain fatty acids as main components of an MCT diet bypass the first step catalyzed by VLCAD and may be readily oxidized by MCAD, supplying the organism with the required energy. It is, therefore, surprising to observe that one year supplementation of the diet does not change the ACAD protein expression in both, WT and VLCAD^−/−^ mice. The oxidation rate towards all used substrates, however, is increased in all analyzed organs of WT mice. VLCAD^−/−^ mice do not reflect such induction although an MCT diet would provide enough substrate for energy production Whereas, it has to be considered that dietary MCFA are mainly elongated and not primarily oxidized [14], [17]–[19]. The marked increase in the turnover rate of C12-CoA in the heart reveals that both, LCAD and MCAD, must be activated by posttranslational modifications as their expression at protein level was unaffected by the diet despite evidence of mitochondrial proliferation in parallel. Very recently, the activity of LCAD has been described to be regulated by deacetylation in a sirtuin mediated process [45]. This regulation is still unknown for MCAD and VLCAD although a constantly growing number of mitochondrial enzymes are described to be regulated in this manner [46], [47]. Based on our results with special respect to WT mice, we suppose that not only LCAD but also MCAD may be activated by deacetylation leading to a synergic activation and resulting in a 30% higher turnover with C12-CoA as substrate in heart homogenate.

In conclusion, we here show that the analyzed organs of one year old VLCAD^−/−^ mice developed different strategies to compensate deficiency of VLCAD. ACAD are not overexpressed at protein level but their contribution to substrate oxidation especially in the liver and heart is impressive. On the other hand, the gastrocnemius muscle undergoes metabolic modification by switch in muscle fibre type reflecting adaptation by enhanced glucose oxidation. Furthermore, the application of an MCT diet over one year has no effect on these strategies, but likely mediates in the heart of VLCAD^−/−^ mice the activation of both, LCAD and MCAD, via posttranscriptional modification.

## Supporting Information

Figure S1 **PPARα expression and catalase activity in WT and VLCAD^−/−^** **mice fed either with LCT or MCT diet.** Tissues tested were (A) liver, (B) heart and (C) skeletal muscle. LCT, long-chain triglyceride; MCT, medium-chain triglyceride; *PPARa,* peroxisome proliferative activated receptor alpha. Values are mean of mean ± SEM (*n* = 5–). * indicates significant differences between WT and VLCAD^−/−^ mice within a diet group. # indicates significant differences between WT or VLCAD^−/−^ mice under different dietary conditions. Values are considered significant if p<0.05 (Two way ANOVA and Student's t-test).
(TIF)

## References

[1] Matsubara Y, Indo Y, Naito E, Ozasa H, Glassberg R, et al (1989) Molecular cloning and nucleotide sequence of cDNAs encoding the precursors of rat long chain acyl-coenzyme A, short chain acyl-coenzyme A, and isovaleryl-coenzyme A dehydrogenases. Sequence homology of four enzymes of the acyl-CoA dehydrogenase family. J Biol Chem 264: 16321–16331. 2777793

[2] Arnold GL, Van HJ, Freedenberg D, Strauss A, Longo N, et al (2009) A Delphi clinical practice protocol for the management of very long chain acyl-CoA dehydrogenase deficiency. Mol Genet Metab 96: 85–90. 19157942 10.1016/j.ymgme.2008.09.008 PMC3219055

[3] Lindner M, Hoffmann GF, Matern D (2010) Newborn screening for disorders of fatty-acid oxidation: experience and recommendations from an expert meeting. J Inherit Metab Dis 33: 521–526. 20373143 10.1007/s10545-010-9076-8

[4] Spiekerkoetter U, Sun B, Zytkovicz T, Wanders R, Strauss AW, et al (2003) MS/MS-based newborn and family screening detects asymptomatic patients with very-long-chain acyl-CoA dehydrogenase deficiency. J Pediatr 143: 335–342. 14517516 10.1067/S0022-3476(03)00292-0

[5] Wilcken B, Wiley V, Hammond J, Carpenter K (2003) Screening newborns for inborn errors of metabolism by tandem mass spectrometry. N Engl J Med 348: 2304–2312. 12788994 10.1056/NEJMoa025225

[6] Mathur A, Sims HF, Gopalakrishnan D, Gibson B, Rinaldo P, et al (1999) Molecular heterogeneity in very-long-chain acyl-CoA dehydrogenase deficiency causing pediatric cardiomyopathy and sudden death. Circulation 99: 1337–1343. 10077518 10.1161/01.cir.99.10.1337

[7] Gregersen N, Andresen BS, Corydon MJ, Corydon TJ, Olsen RK, et al (2001) Mutation analysis in mitochondrial fatty acid oxidation defects: Exemplified by acyl-CoA dehydrogenase deficiencies, with special focus on genotype-phenotype relationship. Hum Mutat 18: 169–189. 11524729 10.1002/humu.1174

[8] Kompare M, Rizzo WB (2008) Mitochondrial fatty-acid oxidation disorders. Semin Pediatr Neurol 15: 140–149. 18708005 10.1016/j.spen.2008.05.008

[9] Spiekerkoetter U, Tokunaga C, Wendel U, Mayatepek E, Exil V, et al (2004) Changes in blood carnitine and acylcarnitine profiles of very long-chain acyl-CoA dehydrogenase-deficient mice subjected to stress. Eur J Clin Invest 34: 191–196. 15025677 10.1111/j.1365-2362.2004.01308.x

[10] Werdich AA, Baudenbacher F, Dzhura I, Jeyakumar LH, Kannankeril PJ, et al (2007) Polymorphic ventricular tachycardia and abnormal Ca2+ handling in very-long-chain acyl-CoA dehydrogenase null mice. Am J Physiol Heart Circ Physiol 292: H2202–H2211. 17209005 10.1152/ajpheart.00382.2006

[11] Exil VJ, Roberts RL, Sims H, McLaughlin JE, Malkin RA, et al (2003) Very-long-chain acyl-coenzyme a dehydrogenase deficiency in mice. Circ Res 93: 448–455. 12893739 10.1161/01.RES.0000088786.19197.E4

[12] Exil VJ, Gardner CD, Rottman JN, Sims H, Bartelds B, et al (2006) Abnormal mitochondrial bioenergetics and heart rate dysfunction in mice lacking very-long-chain acyl-CoA dehydrogenase. Am J Physiol Heart Circ Physiol 290: H1289–H1297. 16199475 10.1152/ajpheart.00811.2005

[13] Goetzman ES, Tian L, Wood PA (2005) Differential induction of genes in liver and brown adipose tissue regulated by peroxisome proliferator-activated receptor-alpha during fasting and cold exposure in acyl-CoA dehydrogenase-deficient mice. Mol Genet Metab 84: 39–47. 15639194 10.1016/j.ymgme.2004.09.010

[14] Primassin S, Tucci S, Herebian D, Seibt A, Hoffmann L, et al (2010) Pre-exercise medium-chain triglyceride application prevents acylcarnitine accumulation in skeletal muscle from very-long-chain acyl-CoA-dehydrogenase-deficient mice. J Inherit Metab Dis 33: 237–246. 20446112 10.1007/s10545-010-9105-7

[15] Spiekerkoetter U, Tokunaga C, Wendel U, Mayatepek E, Ijlst L, et al (2005) Tissue carnitine homeostasis in very-long-chain acyl-CoA dehydrogenase-deficient mice. Pediatr Res 57: 760–764. 15774826 10.1203/01.PDR.0000157915.26049.47

[16] Spiekerkoetter U, Ruiter J, Tokunaga C, Wendel U, Mayatepek E, et al (2006) Evidence for impaired gluconeogenesis in very long-chain acyl-CoA dehydrogenase-deficient mice. Horm Metab Res 38: 625–630. 17075770 10.1055/s-2006-954581

[17] Tucci S, Primassin S, Ter VF, Spiekerkoetter U (2010) Medium-chain triglycerides impair lipid metabolism and induce hepatic steatosis in very long-chain acyl-CoA dehydrogenase (VLCAD)-deficient mice. Mol Genet Metab 101: 40–47. 20580297 10.1016/j.ymgme.2010.05.005

[18] Tucci S, Primassin S, Spiekerkoetter U (2010) Fasting-induced oxidative stress in very long chain acyl-CoA dehydrogenase-deficient mice. FEBS J 277: 4699–4708. 20883455 10.1111/j.1742-4658.2010.07876.x

[19] Tucci S, Flogel U, Sturm M, Borsch E, Spiekerkoetter U (2011) Disrupted fat distribution and composition due to medium-chain triglycerides in mice with a beta-oxidation defect. Am J Clin Nutr 94: 439–449. 21697078 10.3945/ajcn.111.012948

[20] Bradford MM (1976) A rapid and sensitive method for the quantitation of microgram quantities of protein utilizing the principle of protein-dye binding. Anal Biochem 72: 248–254. 942051 10.1016/0003-2697(76)90527-3

[21] Mohanty JG, Jaffe JS, Schulman ES, Raible DG (1997) A highly sensitive fluorescent micro-assay of H2O2 release from activated human leukocytes using a dihydroxyphenoxazine derivative. J Immunol Methods 202: 133–141. 9107302 10.1016/s0022-1759(96)00244-x

[22] Zhou M, Diwu Z, Panchuk-Voloshina N, Haugland RP (1997) A stable nonfluorescent derivative of resorufin for the fluorometric determination of trace hydrogen peroxide: applications in detecting the activity of phagocyte NADPH oxidase and other oxidases. Anal Biochem 253: 162–168. 9367498 10.1006/abio.1997.2391

[23] Ter VF, Mueller M, Kramer S, Haussmann U, Herebian D, et al (2009) A novel tandem mass spectrometry method for rapid confirmation of medium- and very long-chain acyl-CoA dehydrogenase deficiency in newborns. PLoS One 4: e6449. 19649258 10.1371/journal.pone.0006449 PMC2715108

[24] Schafer C, Hoffmann L, Heldt K, Lornejad-Schafer MR, Brauers G, et al (2007) Osmotic regulation of betaine homocysteine-S-methyltransferase expression in H4IIE rat hepatoma cells. Am J Physiol Gastrointest Liver Physiol 292: G1089–G1098. 17218476 10.1152/ajpgi.00088.2006

[25] Lin J, Wu H, Tarr PT, Zhang CY, Wu Z, et al (2002) Transcriptional co-activator PGC-1 alpha drives the formation of slow-twitch muscle fibres. Nature 418: 797–801. 12181572 10.1038/nature00904

[26] Michael LF, Wu Z, Cheatham RB, Puigserver P, Adelmant G, et al (2001) Restoration of insulin-sensitive glucose transporter (GLUT4) gene expression in muscle cells by the transcriptional coactivator PGC-1. Proc Natl Acad Sci U S A 98: 3820–3825. 11274399 10.1073/pnas.061035098 PMC31136

[27] Gelinas R, Thompson-Legault J, Bouchard B, Daneault C, Mansour A, et al (2011) Prolonged QT interval and lipid alterations beyond beta-oxidation in very long-chain acyl-CoA dehydrogenase null mouse hearts. Am J Physiol Heart Circ Physiol 301: H813–H823. 21685264 10.1152/ajpheart.01275.2010 PMC3191095

[28] Primassin S, Ter VF, Mayatepek E, Spiekerkoetter U (2008) Carnitine supplementation induces acylcarnitine production in tissues of very long-chain acyl-CoA dehydrogenase-deficient mice, without replenishing low free carnitine. Pediatr Res 63: 632–637. 18317232 10.1203/PDR.0b013e31816ff6f0

[29] Gu Z, Steinmetz LM, Gu X, Scharfe C, Davis RW, et al (2003) Role of duplicate genes in genetic robustness against null mutations. Nature 421: 63–66. 12511954 10.1038/nature01198

[30] Maslov S, Sneppen K (2002) Specificity and stability in topology of protein networks. Science 296: 910–913. 11988575 10.1126/science.1065103

[31] Lefaucheur L (2010) A second look into fibre typing--relation to meat quality. Meat Sci 84: 257–270. 20374784 10.1016/j.meatsci.2009.05.004

[32] Pette D, Staron RS (2000) Myosin isoforms, muscle fiber types, and transitions. Microsc Res Tech 50: 500–509. 10998639 10.1002/1097-0029(20000915)50:6<500::AID-JEMT7>3.0.CO;2-7

[33] Quiroz-Rothe E, Rivero JL (2004) Coordinated expression of myosin heavy chains, metabolic enzymes, and morphological features of porcine skeletal muscle fiber types. Microsc Res Tech 65: 43–61. 15570587 10.1002/jemt.20090

[34] Schiaffino S, Reggiani C (1994) Myosin isoforms in mammalian skeletal muscle. J Appl Physiol 77: 493–501. 8002492 10.1152/jappl.1994.77.2.493

[35] Benard G, Faustin B, Passerieux E, Galinier A, Rocher C, et al (2006) Physiological diversity of mitochondrial oxidative phosphorylation. Am J Physiol Cell Physiol 291: C1172–C1182. 16807301 10.1152/ajpcell.00195.2006

[36] Forner F, Foster LJ, Campanaro S, Valle G, Mann M (2006) Quantitative proteomic comparison of rat mitochondria from muscle, heart, and liver. Mol Cell Proteomics 5: 608–619. 16415296 10.1074/mcp.M500298-MCP200

[37] Mootha VK, Bunkenborg J, Olsen JV, Hjerrild M, Wisniewski JR, et al (2003) Integrated analysis of protein composition, tissue diversity, and gene regulation in mouse mitochondria. Cell 115: 629–640. 14651853 10.1016/s0092-8674(03)00926-7

[38] Glatz JF, Jacobs AE, Veerkamp JH (1984) Fatty acid oxidation in human and rat heart. Comparison of cell-free and cellular systems. Biochim Biophys Acta 794: 454–465. 6430348 10.1016/0005-2760(84)90012-2

[39] Wang H, Hiatt WR, Barstow TJ, Brass EP (1999) Relationships between muscle mitochondrial DNA content, mitochondrial enzyme activity and oxidative capacity in man: alterations with disease. Eur J Appl Physiol Occup Physiol 80: 22–27. 10367719 10.1007/s004210050553

[40] Chegary M, Brinke H, Ruiter JP, Wijburg FA, Stoll MS, et al (2009) Mitochondrial long chain fatty acid beta-oxidation in man and mouse. Biochim Biophys Acta 1791: 806–815. 19465148 10.1016/j.bbalip.2009.05.006 PMC2763615

[41] Lea W, Abbas AS, Sprecher H, Vockley J, Schulz H (2000) Long-chain acyl-CoA dehydrogenase is a key enzyme in the mitochondrial beta-oxidation of unsaturated fatty acids. Biochim Biophys Acta 1485: 121–128. 10832093 10.1016/s1388-1981(00)00034-2

[42] He M, Rutledge SL, Kelly DR, Palmer CA, Murdoch G, et al (2007) A new genetic disorder in mitochondrial fatty acid beta-oxidation: ACAD9 deficiency. Am J Hum Genet 81: 87–103. 17564966 10.1086/519219 PMC1950923

[43] Nouws J, Nijtmans L, Houten SM, van den BM, Huynen M, et al (2010) Acyl-CoA dehydrogenase 9 is required for the biogenesis of oxidative phosphorylation complex I. Cell Metab. 12: 283–294. 10.1016/j.cmet.2010.08.002 20816094

[44] Handschin C, Spiegelman BM (2008) The role of exercise and PGC1alpha in inflammation and chronic disease. Nature 454: 463–469. 18650917 10.1038/nature07206 PMC2587487

[45] Hirschey MD, Shimazu T, Goetzman E, Jing E, Schwer B, et al (2010) SIRT3 regulates mitochondrial fatty-acid oxidation by reversible enzyme deacetylation. Nature 464: 121–125. 20203611 10.1038/nature08778 PMC2841477

[46] Choudhary C, Kumar C, Gnad F, Nielsen ML, Rehman M, et al (2009) Lysine acetylation targets protein complexes and co-regulates major cellular functions. Science 325: 834–840. 19608861 10.1126/science.1175371

[47] Zhao S, Xu W, Jiang W, Yu W, Lin Y, et al (2010) Regulation of cellular metabolism by protein lysine acetylation. Science 327: 1000–1004. 20167786 10.1126/science.1179689 PMC3232675

